# Enhanced Ultra-Sensitive Metamaterial Resonance Sensor based on Double Corrugated Metal stripe for Terahertz Sensing

**DOI:** 10.1038/s41598-019-44026-4

**Published:** 2019-05-17

**Authors:** Sajad Niknam, Mehran Yazdi, Salman Behboudi Amlashi

**Affiliations:** 1 Department of Computer Science & Engineering and Information Technology, Shiraz University, Shiraz, Iran; 2 Department of Communications & Electronics, School of Electrical and Computer Engineering, Shiraz University, Shiraz, Iran; 3 Department of Electrical Engineering, Shahed University, Tehran, Iran

**Keywords:** Imaging and sensing, Biomedical engineering, Imaging and sensing, Biomedical engineering

## Abstract

In this paper, an ultra-sensitive metamaterial terahertz sensor is proposed. The resonance sensor is designed based on a novel double corrugation form to enhance the ability of the sensor in the terms of sensitivity, Q-factor and the maximum sensible thickness of an analyte. The introduced structure can support the spoof surface plasmon and can resonate strongly at the tuned frequencies. Moreover, the structure of the terahertz sensor is investigated thoroughly from different points of view including frequency shifts due to variations in the thickness or refractive index of the analyte. In addition, the sensitivity of the sensor is approximated with a biharmonic fitting function for different combinations of refractive index and analyte thickness as “sensitivity surface”. The sensor shows the maximum sensitivity of 1.75 THz/RIU for refractive index between 1–1.2 with a maximum thickness of 80 μm. Moreover, the simulation results approved that the double corrugation on the metal stripe improves the electromagnetic field interaction in the metal part greatly in comparison with the previously reported works. According to this work, the proposed structure can be applied for terahertz sensing with more abilities to sense even thicker biologic tissues.

## Introduction

In recent decades, many researches have been done in the field of terahertz and many applications have been emerged through several investigation^1,2^. Accordingly, due to special interaction of the terahertz wave with biologic materials, biomedical applications of THz radiation are very much considered^3–7^. In fact, the low photon energy of THz spectra wave has made it very attractive in biological imaging and spectroscopy to the extent that many researchers have tried to provide new and more effective methods for synthesizing THz radiation based on these applications^8–14^. Recently, the sensing of the biologic material in the THz spectra has turned to a fascinating topic of research. Therefore, engineers and scientists examined novel methods for the identification of different analyte by THz spectroscopy including approaches based on transmission, absorption, shifts in frequency responses, refractive index sensing, or variations of phase^15–22^. Xie *et al*. applied an array of highly sensitive square-shaped slits on a silicon substrate for terahertz transmission spectroscopy of antibiotics at 0.3 THz^23^. In a similar approach, a Nano-slot-antenna array-based THz sensing method is used for the discrimination of carbohydrates in the frequency range between 0.5–2.5 THz based on both absorption and transmission spectra^24^. One of the most interesting techniques in terahertz sensing is the frequency shift sensing by which the shifts in the resonance modes of a transmission spectra of a structure is measured for the detection of the changes in the analyte characteristics^25–31^. Interestingly, the metallic nanotrenches coupled with water is reported as a high contrast terahertz sensor that can be applied to a various types of polar organic molecules based on resonant frequency shifts^32^. Although, in the mentioned report, resonant frequencies are appeared in sub THz frequencies that makes sensitivity parameter limited for the frequency shift detection. On the other hand, for such structures, there are significant losses due to attenuation in the terahertz regime as well as low confinement of electromagnetic field with the structure^33^. Due to these losses, the Q-factor of the resonant modes has low values in several reported structures. Thus, the electromagnetic interaction with metals cannot be strong enough to cause sharp resonance dips in their structures. Contrary to what occurs in terahertz frequencies, surface plasmon polariton (SPP) sensing demonstrates tightly confined local field that brings about higher Q-factors and sensitivity at very higher frequencies (visible wavelength)^34–37^ with noble metals such as silver and gold^38^. Conversely, these noble metals cannot bind the THz electromagnetic field at its surface because of its dimensions relative to the terahertz wavelength scale. Although it is not possible to use such a feature in terahertz structures but many researches proved that corrugated metal structures (even with noble metals) could support surface waves greatly that can improve the confinement of local electromagnetic field at the surface boundary of metals. These surface waves, named spoof surface plasmon (SSP) waves, can mimic the properties of SPPs in terahertz spectrum^39–41^. Because of SSP modes, tightly confined local fields can be observed and due to this, much different types of terahertz sensors are proposed based on metamaterial structures^42–45^. The eye-catching characteristics of metamaterial structure in controlling the electromagnetic field make it a solution for improving the field confinement in terahertz sensors. In this paper, a terahertz sensor is investigated based on metamaterial structure that its unit cell consists of a corrugated metal stripe. A novel corrugation form, as is called double corrugation, is perforated on the metal part and the obtained results show great improvement compared to similar reported works^46^. In addition, the sensitivity and the Q-factor of the SSP resonance modes are reported in this paper and are assessed statistically. The proposed structure can be successfully applied for terahertz sensing for the variations in refractive index and thickness of an analyte. It is worth mentioning that the electric field and surface current of the structure, which are depicted later, indicate a significant betterment than similar structures. As result, this improvement causes higher sensitivity as well as more ability for sensing thicker analyte.

## Structure Design

**Figure 1:**
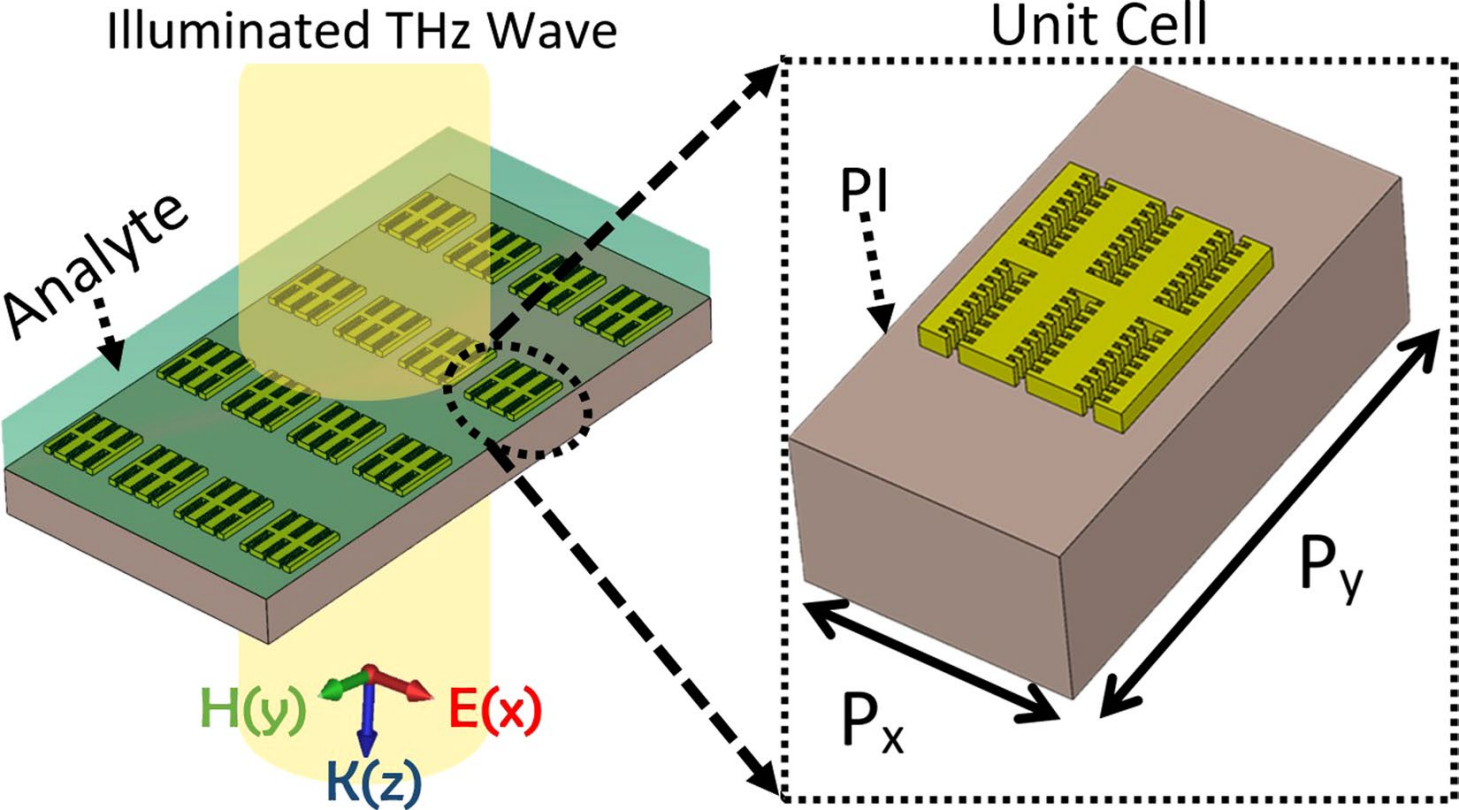
The structure of the proposed metamaterial sensor with the illumination of THz beams. The analyte is superimposed on the sensor structure, which causes an environmental change in refractive index and consequently, shifts the resonance frequency. The unit cell of metamaterial structure has the dimensions P_y_ = 68 μm and P_X_ = 24 μm. The PI layer has a thickness equals to 15 μm.

A novel structure of metamaterial terahertz sensor is proposed in Fig. 1. As depicted, the metamaterial structure consists of a unit cell with metal stripe, which is grooved in two sides and form symmetrical metal arms. Moreover, each arm is corrugated in two different orders but with the same intervals on the edges. The metallic structure is placed on a layer of polyimide substrate. The polyimide layer is transparent for these frequencies due to its low loss tangent of 0.005 with a dielectric constant 3 in these wavelengths^47^. With this corrugation form on the metal stripe, it is expected that the proposed structure can support spoof surface plasmon modes, which can be considered in dispersion diagram of the structure in the next section.

**Figure 2:**
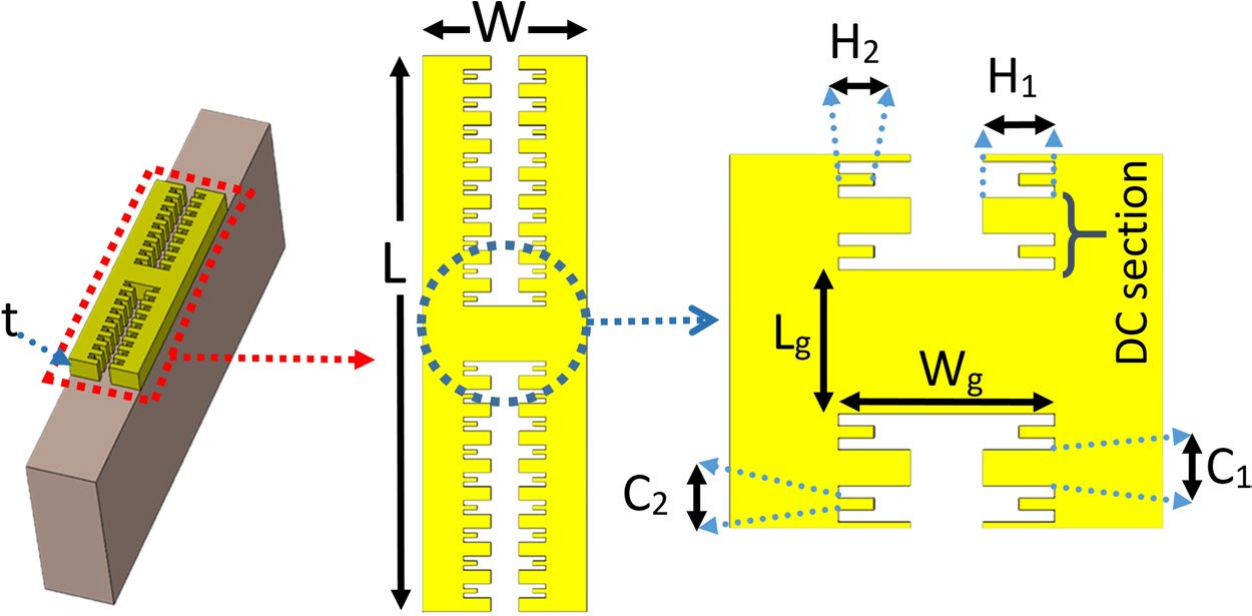
The unit cell of the metamaterial structure is formed from a periodic H-shaped metal which supports surface wave due to its corrugations. The dimensions are included W = 6 μm, Lg = 2 μm, Wg = 3 μm, H1 = 2*H2 = 1 μm, C1 = 3*C2 = 0.5 μm and DC section of 1 μm that through the simulation are fixed for this period. Also, t is the thickness of metal stripe and is equal to 2 μm. The size L can be tuned for the desired resonance frequency.

As Fig. 1 Represents the structure, the dimensions P_y_ and P_x_ of the unit cell are 68 μm and 24 μm for a metal stripe with L = 26 μm. With a closer look at the dimensions of the metal structure, it can be noted that L is the length of the metal stripe as can be seen in Fig. 2. In fact, the metamaterial structure is formed from a periodic unit cell in directions x and y with periodicities P_x_ and P_y_ respectively. In each unit cell, the metal part is originated from three repeating H-shaped metal stripes that stick together and one period is shown individually in Fig. 2. The width of each repeating H-shaped metal stripe is constant through this paper and is W = 6 μm. The length of metal stripe is L that can be tuned for different resonance frequencies. In addition to those, the gap sizes between metal arms, i.e., L_g_ and W_g_, have fixed values of 2 μm and 3 μm respectively. The other dimensions in the structure of the metal stripe such as C_1_ and C_2_, are the widths of toothed–shaped modifications and have the sizes equal to C_1_ = 3C_2_ = 0.5 μm. It is necessary to state that the length of C_2_ is calculated for best possible results. In addition, H_1_ and H_2_ are the lengths of the tooth-shaped modifications. As aforementioned, the inner edges in each metal arm are modified in two ways, first, they are corrugated with periodic grooves equidistantly with periodicity of 1 μm and Besides, with a similar periodicity, they are toothed in the middle of the primary corrugated grooves as depicted in Fig. 2, which altogether form a double-corrugation (DC) shape. As it is illustrated in Fig. 2, each metal arm supports an equal number of periodic DC sections. By changing the number of these periodic DC sections, we can tune the resonance frequencies of the structure.

## Results and Discussion

**Figure 3:**
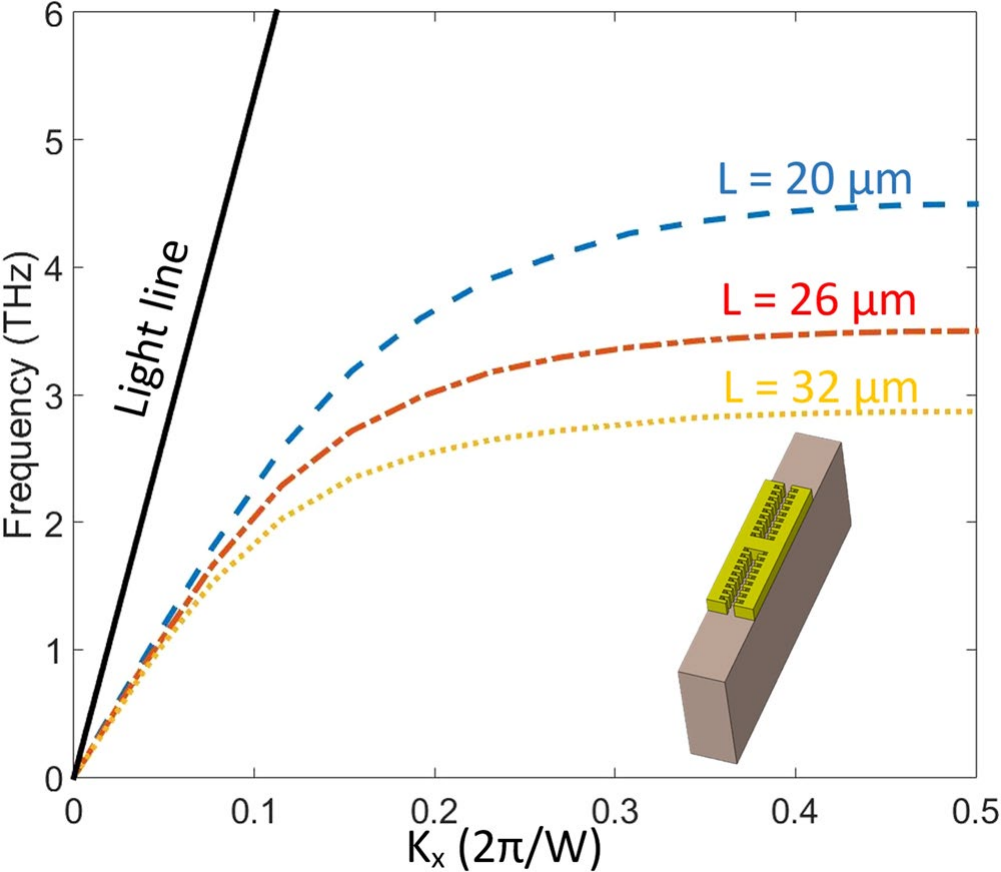
The dispersion diagram plotted for one period of corrugated metal stripe. For different sizes of the metal stripe (L), dispersion curve asymptote to a specific frequency of SSP mode. The dispersion diagram proves that the interaction of electromagnetic field with metal has similar behavior to surface plasmon polariton in visible wavelength. Increasing L causes a decrease in the asymptote frequency. There is a linear relation between frequency and wavenumber for the light line (black solid line) in the PI substrate.

**Figure 4:**
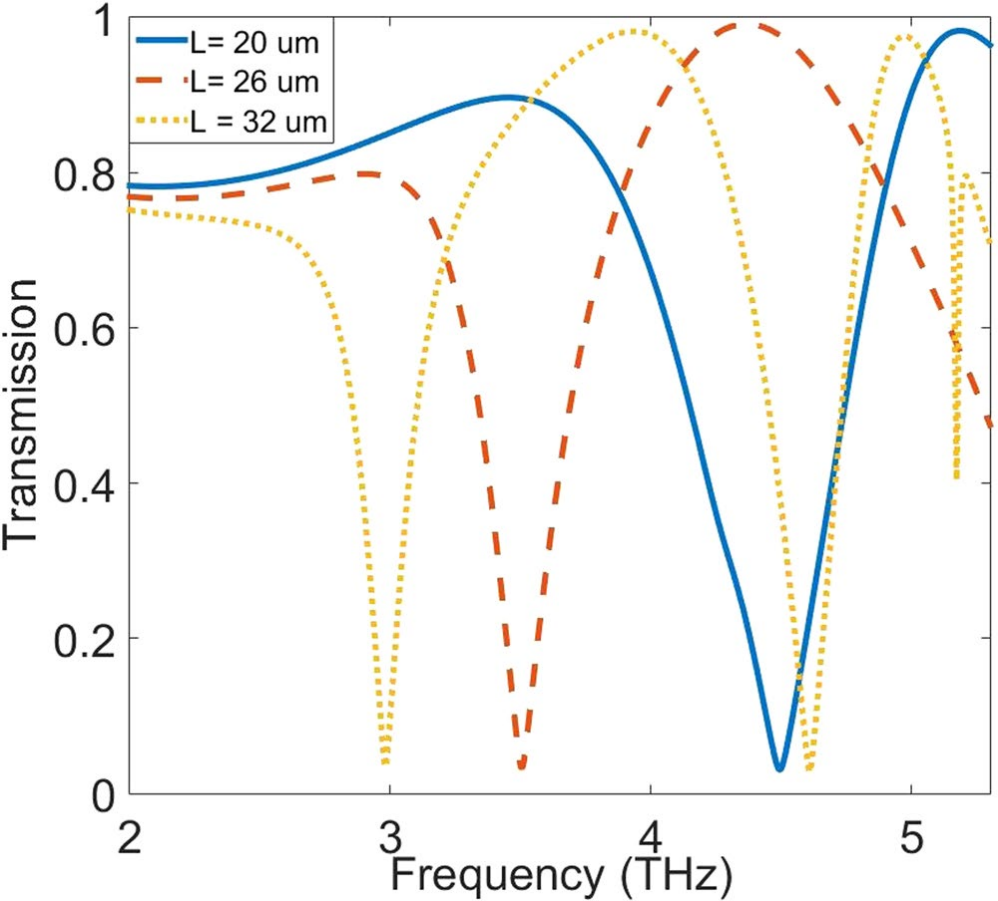
Spectral Transmission diagram of the metamaterial structure. The transmission curves have redshifts when L is increased. For each L, the transmission curve shows multiple resonance dips. Due to corrugated form of metal structure, the resonances have Fabry-Perot form, which expresses the sensing ability.

Considering the dispersion diagram of the structure in Fig. 3, it gives rise to this fact that for different sizes of L, there are correspondent asymptote frequencies. As matter of fact, increasing the wavenumber causes the dispersion curve asymptote to a certain frequency despite the linear relation of the light line (see black solid line in Fig. 3) in the substrate (PI). Such non-linear and asymptotic behavior in dispersion curves imitates the surface plasmon polariton in optical frequencies, which approves that the structure supports surface waves. The dispersion curves of the unit cell show highly localized SSP modes on the surface of the structure for each asymptote frequency. In Fig. 3, it is also noteworthy that with increasing the length of L, the asymptotic frequency of the SSP modes decreases. In Fig. 4, the transmission spectra curves are plotted for different sizes of metal stripe (L). The transmission diagram calculated for three sizes L = 20 μm, 26 μm and 32 μm which correspond to 9, 12 and 15 DC sections respectively for each side arm. By increasing the DC sections, as illustrated in Fig. 4, a redshift occurs in the resonance dips. As calculated for each size, there are two main resonance dips, which form Fabry-Perot (FP) resonance shape at their resonance frequencies. Hence, these FP-shaped resonances can justify the sensing performance of the structure in terahertz frequencies. In Fig. 4, for the simplicity, only first resonances that are in match with asymptote SSP-mode frequencies of their dispersion diagram are shown. As an important parameter for sharp resonances, the Q-factor of each dip in transmission diagram quantifies its capability for sensing. Multiple resonances are observed in the transmission spectra diagram with different Q-factors for different sizes of metal stripe (or different number of DC sections). In the Table 1, the Q-factors of two first resonances of the transmission diagram are calculated for three different sizes. The calculated Q-factors show great improvement and qualification compared to the similar reported structures. Generally, SSP modes are very sensitive to the dielectric constant of their environment as well as their variations. In this case, each parameter that can change the surrounding dielectric environment can have an effect on the resonance frequencies of the structure such as refractive index (RI) and analyte thickness^48^.

**Table 1:** Q-factors for different sizes of metal stripe are calculated for the first and the second resonances of the transmission.

L size (DC sections in each side arm)	1^st^ resonance Q-factors	2^nd^ resonance Q-factors
20 μm (9)	172.7	301
26 μm (12)	195.2	237
32 μm (15)	161.5	347

**Figure 5:**
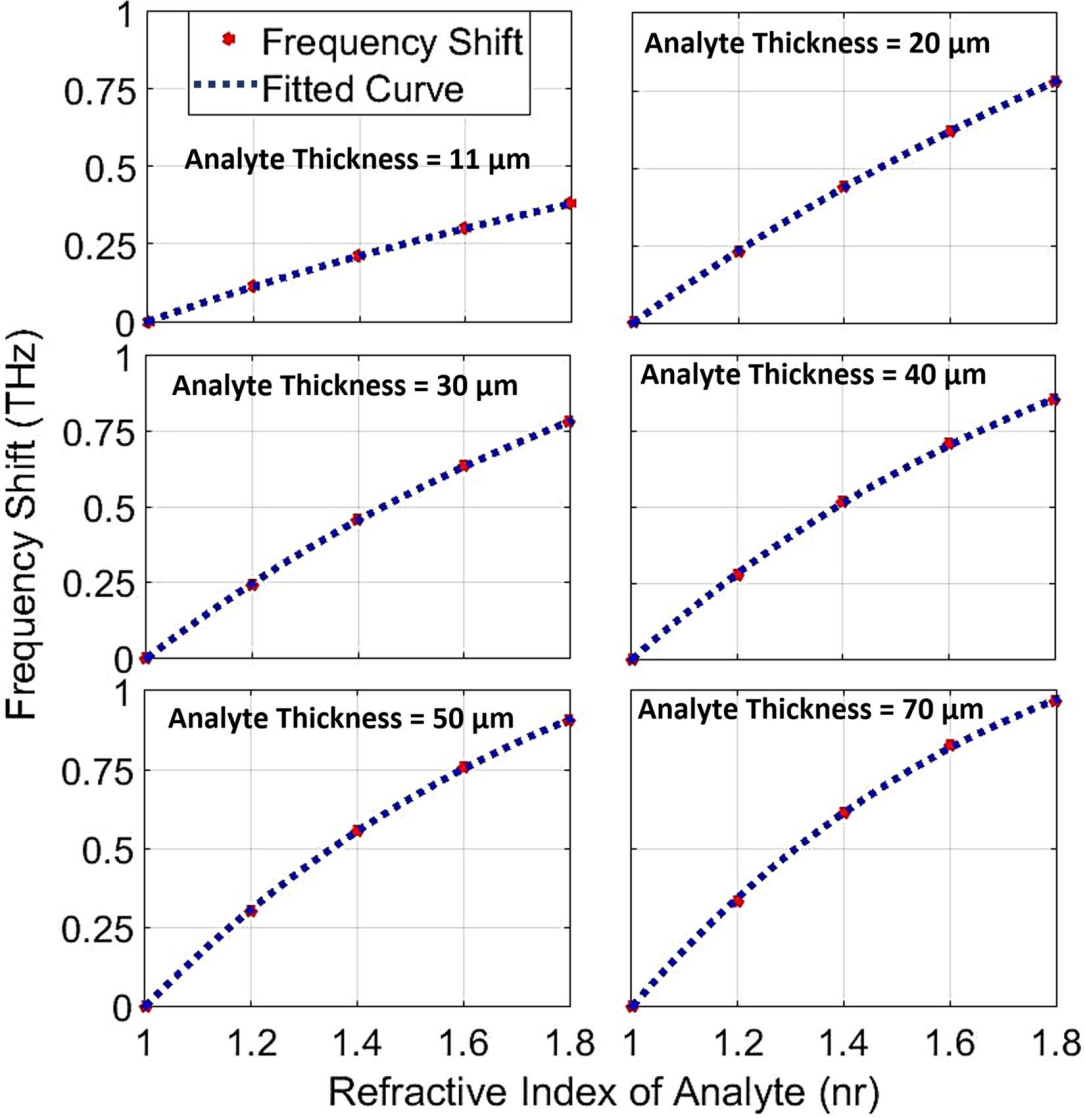
Frequency shifts for different refractive indices are plotted and estimated by the fitting function. For multiple analyte thicknesses, the diagram depicts frequency shifts. Using the fitting curve, it is possible to predict frequency shifts for other values of RI. The range of RI corresponds to most important refractive indices in biological tissues.

**Figure 6:**
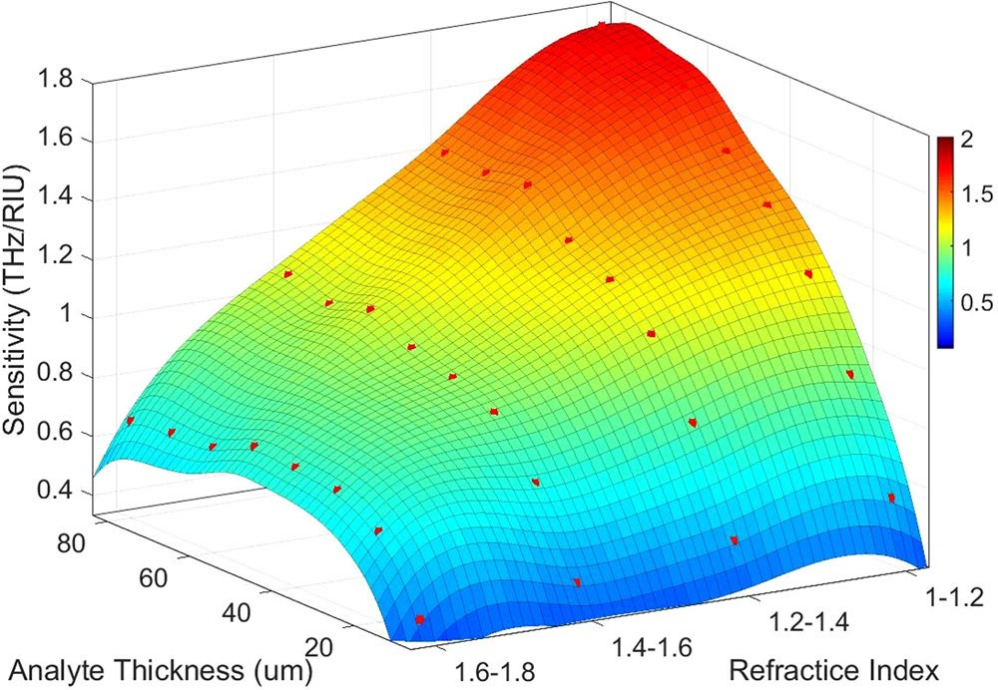
Sensitivity of the sensor according to both the thickness and the refractive index of the analyte as sensitivity surface. The diagram shows that there are different sensitivities when thickness and refractive index of the analyte change. By examining this three-dimensional surface, a more accurate analysis of the relationship between sensitivity of the analyte parameters such as refractive index and thickness can be obtained. The red dots are the samples come from the simulations and the surface is estimated with a biharmonic fitting function. As a result, this chart helps to obtain a more correct choice of dimension values for different sensing applications.

**Figure 7:**
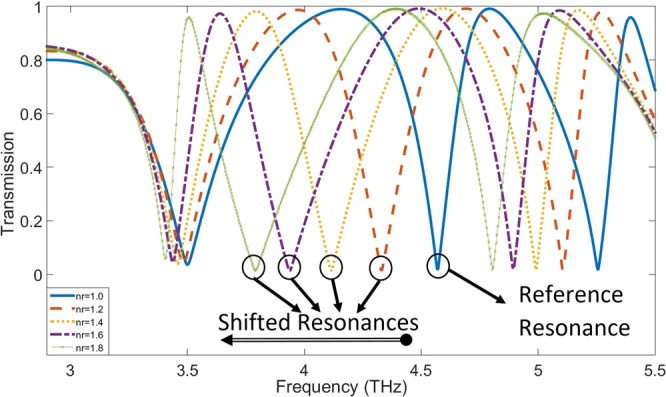
Transmission diagram for an analyte with different RIs. The analyte with RI of one is considered as reference resonance and other shifted resonances are compared with it. As depicted, with increasing the RI a redshift happens for the reference resonance. The second resonance is applied for sensing because of better sensing features.

**Figure 8:**
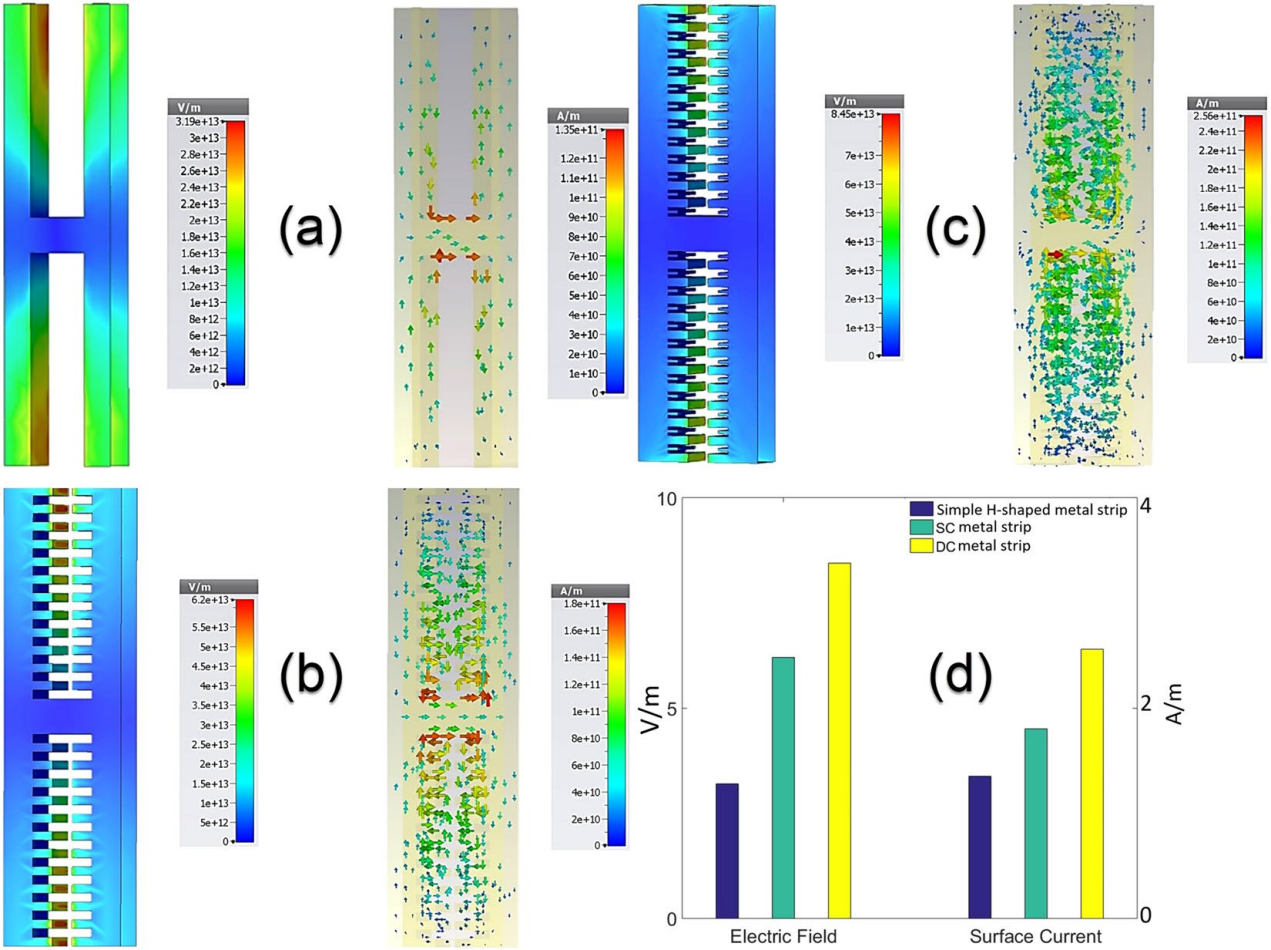
Analysis of electric parameters for different structures, which can be used as the indicators of sensing ability. (**a**) A simple H-shaped structure without corrugation is investigated for electric field distribution and its surface current. Maximum electric field cannot reach more than 3.19e + 13 V/m on the top of the edges. The maximum surface current is 1.35e + 11 A/m at its highest possible value. (**b**) Due to the single corrugations (SC) that are created on the inner edges, the maximum electric field is about 2 times of the former structure and the surface current has 30 percent increase. (**c**) final proposed structure has double corrugation (DC) form on its inner edges and the maximum electric field is enhanced nearly 36 percent better than previous structure in (**b**) and also the surface current meets a growth of 44 percent relative to SC form. (**d**) In the bar chart, the maximum distributed electric fields and surface currents of three structures are compared for better analysis.

The refractive index (RI) of analyte can change the resonance frequency in a meaningful manner. The frequency shifts that occur due to changes in the analyte thickness are calculated and plotted for different RIs in Fig. 5. The range of RI in these diagrams covers values between 1 to 1.8 because of the importance of these refractive indices in biological spectroscopy^49–51^. These frequency shifts, is obtained by a sensor structure with L = 26 μm and furthermore, the second resonance of the transmission spectra diagram with Q-factor of 237 is applied as the reference resonance. Also, the FS-RI data in Fig. 5 is approximated with a curve-fitting function f(x) = a.exp(−x) + b.x + c as a best fit where the fitting coefficients are introduced in Table 2. These coefficients show that the FS for this structure has an exponential behavior alongside of its linear nature. To estimate the proper fitting curves, different linear and non-linear fitting functions are examined to reach the best fit. The primary estimation for the curves is a linear increase based on calculated FS data. As can be clearly seen, the overall trend of calculated FSs experience an almost positive slope in each incremental step of RIs in the ideal form. Therefore, a linear expression is simply predictable. On the other hand, the analysis of electric field and surface current on the structure shows a saturation state in these parameters that can be modeled with an exponential decay term. In addition, a constant is added to fine-tuning the fitting function. According to the Fig. 5, for a certain analyte thickness, frequency shifts follow the fitting function in a defined range of refractive index. Furthermore, for the thicker analyte, the shifts become greater. The sensitivity of the terahertz sensor, as an important Figure of merit, can distinguish this structure from the previously reported works. The sensitivity has a direct relation with Q-factor of the resonances and as the results have shown that the proposed structure demonstrates a great improvement in its Q-factor originating from sharp resonance dips in the transmission spectra. The sensitivity of the sensor is defined here by the expression (FS_1_ − FS_0_)/ (nr_1_ − nr_0_) in which FS_1_ and FS_0_ are the frequency shifts that occur due to change in analyte RI from one to nr_1_ and nr_0_ respectively. In the mentioned expression, each nr corresponds to frequency shift FS which is calculated in proportion to reference resonance frequency. According to the outcome of the simulations, it is found that the sensitivity of the structure for a fixed analyte thickness depends on the refractive index of the analyte. Therefore, there are multiple sensitivities based on changes in RI and thickness of analyte as it is concluded in Fig. 6. Here, according to the data obtained from simulations, RIs of analyte are classified into four categories. Based on simulation results, the sensitivity changes when the value of RI alters between different classes. For this reason, it is not possible to determine an exact value for the sensitivity of the sensor and therefore a “sensitivity surface” is defined for the prediction of sensor behavior. For further explanation of Fig. 6, it should be noted that red dots are from the simulated data and the colored surface is estimated using a biharmonic fitting function. As can be deduced, a maximum value of 1.75 THz/RIU is achievable which is very high sensitivity in all similar previous works for such analyte thickness. Another acquirable result from the Fig. 6 is the maximum analyte thickness before that the sensitivity reaches to its maximum value (saturation state). For a more detailed explanation, it is worth noting that the saturation state is defined here as a state that there is no growth in sensitivity with increase in the analyte thickness for a constant refractive index of analyte. Another important point that should be noticed is that there are different “maximum sensible thicknesses” for different analyte refractive indices. Referencing the results in Fig. 6, for analyte with 1 < RI < 1.2 there is a constant growth with increase in the analyte thickness. In other word, as much the analyte is thicker the sensitivity would be higher and up to these results, it can be thick to the maximum 80 μm with sensitivity nearly 1.75 THz/RIU. Following the results, for analyte with 1.2 < RI < 1.4 there is an decrease in the maximum sensitivity to 1.42 THz/RIU at the maximum sensible analyte thickness close to 70 μm. This class of materials are very important because they have very close RI to water that is the main constituent substance of many organic and biological tissues in THz frequencies^52–54^. Moreover, for the analyte with 1.4 < RI < 1.6, the maximum sensitivity that is achievable reaches to 1.1 THz/RIU for the thickness of 70 μm. Also, in the range of 1.6 < RI < 1.8, a maximum sensitivity attains the value of 0.75 THz/RIU at analyte thickness of 50 μm. According to these results, it is possible to define a range of sensitivity for the sensor about 0.4–1.75 THz/RIU, which actually includes analyte thickness between 10 μm and 80 μm for variations of RI in the range of 1–1.8. In Fig. 7, the frequency shifts in transmission diagram of the structure are shown. A structure with size L equals to 26 μm (12 DC sections) is applied for sensing an analyte with thickness of 30 μm. RI of analyte is increased from 1 to 1.8 where steps equal to 0.2. It can be seen that the second resonance of transmission diagram has redshifts due to increase in the refractive index of the analyte. Moreover, there are frequency shifts in third resonance (higher order mode) but because the second resonance has better Q-factor as well as higher frequency shifts, it is more suitable for sensing purpose. The results give rise to the belief that there must be a strong electric field and surface current on the structure. The maximum analyte thickness determines that stronger SSP modes are stimulated in this structure, compared to the results obtained by previously reported structures^25,26^. Hence, to illustrate the novelty of this work, three different structures have been investigated in this regard. Hereby, three similar structures with double corrugation (DC), simple corrugation (SC) and simple H-shaped metal stripe (H-shaped) are considered. Figure 8 compares these three structures with each other very well. In Fig. 8a, the first structure is a simple H-shaped metal stripe without any corrugation in its each of side arms. The maximum value for electric field happens only on the end of the each arm with magnitude of 3.9e + 13 V/m. In addition, the surface current in this structure has the maximum magnitude of 1.35e + 11 A/m. Next, in a similar structure, a corrugation has been created on the edge of each metal arm as illustrated in Fig. 8b. Due to more confined electric field between corrugations and formation of Fabry-Perot sharp resonances, the electric field generated in the structure, is nearly 2 times of the aforementioned structure. In a same way, a 30 percent increase is occurred to surface current. Based on the results shown in Fig. 8c, the proposed structure is introduced with double corrugation form that excites a greater field and surface current than previous formations. The second corrugations, which are created in between the first corrugations, must be smaller than the first corrugations.

**Table 2:** The coefficients of the fitting curve for different analyte thicknesses.

Analyte Thickness (µm)	Fitting Coefficients (f(x) = a.exp(−x) + bx + c)		
	a	b	c
11	−1.084	0.2	0.198
20	−1.932	0.292	0.417
30	−3.225	0.165	1.02
40	−4.293	−0.010	1.587
50	−5.05	−0.140	1.995
70	−6.5	−0.413	2.78

The simulations approved that the second corrugations must be in the range of 0.3H_1_ < H2 < 0.8H_1_ otherwise there is a decrease in the both field and surface current. For this range, the results are similar to the time when H_2_ = 0.5H_1_, which is considered in this paper. As the result of the comparison of the three structures, Fig. 8d shows the conclusion in a bar graph. It is comparable that the proposed structure has improved the electric field and surface current greatly relative to other ones and statistically, its maximum electric field reaches to more than 2.6 times greater than the first structure. Furthermore, the surface current has also similar condition in DC structure and is nearly two times of simple H-shaped metallic stripe.

## Method

It is necessary to state that the simulation results are thoroughly obtained by CST suite-2018 as numerical tools. For the dispersion curve, the boundary condition in x and y direction is considered as periodic and the electric boundary condition (E_t_ = 0) is applied for z-direction. Then the phase variation in x-direction is swept from 0 to 180 degree. The transmission curves, are obtained from the response of the structure to an illuminated electromagnetic wave while the excitation wave is considered as Floquet port mode with 2 essential modes. In addition, the curve fitting and surface fitting is done by Matlab 2015 software. The curve fitting is based on an exponential and a linear terms and for the surface fitting, a biharmonic function is used to achieving the best possible estimation. Finally, the gold is chosen as metal for all the simulations.

## Data Availability

The simulation results generated by CST and Matlab during the current study are available from the corresponding author on reasonable request.
